# Supplementary material for: Beyond facility-based births: Is Uganda delivering effective maternal and newborn care? An analysis of the 2022 demographic health survey and 2023 harmonized health facility assessment survey
Source: PLOS Glob Public Health. 2025 Oct 30;5(10):e0004949. doi: 10.1371/journal.pgph.0004949 (PMC12574829; doi:10.1371/journal.pgph.0004949)
Supplement: S1 Table — (DOCX) [file pgph.0004949.s001.docx]

**S1 Table: Harmonisation of regions and facility levels for linking DHS and HHFA data sets**

| **Harmonized Region category** | **UDHS region categories** | **HHFA region categories** |
| --- | --- | --- |
| Kampala | Kampala | Kampala |
| Central | Buganda | South central  North Central |
| Karamoja | Karamoja | Karamoja |
| Mid-North | Acholi, Lango | Acholi, Lango |
| Mid-East | Busoga, Elgon | Busoga, Bugisu |
| North East | Bukedi, Teso | Bukedi, Teso |
| Mid-west | Bunyoro, Tooro | Bunyoro, Tooro |
| South-West | Ankole, Kigezi | Ankole, Kigezi |
| West Nile | West Nile | West Nile |
| **Health Facility Level** | | |
| **Harmonized Facility category** | **UDHS facility categories** | **HHFA facility categories** |
| Government hospital | Government hospital | National or Regional referral Hospital  General Hospital |
| Government Health Centre | Government Health Centre  Other Public sector | HCIV  HCIII  HCII |
| Private Facility | Private Hospital/clinic  Other private sector  other | Private health practice  (Private not for Profit, Private for Profit, Non-Governmental Organization, Faith Based Organization) |

HC: Health center

DHS: Demographic Health Survey

UDHS: Uganda Demographic Health Survey

HHFA: Harmonized Health Facility Assessment
