# Supplementary material for: Beyond facility-based births: Is Uganda delivering effective maternal and newborn care? An analysis of the 2022 demographic health survey and 2023 harmonized health facility assessment survey
Source: PLOS Glob Public Health. 2025 Oct 30;5(10):e0004949. doi: 10.1371/journal.pgph.0004949 (PMC12574829; doi:10.1371/journal.pgph.0004949)
Supplement: S2 Table — (DOCX) [file pgph.0004949.s002.docx]

**S2 Table**: **indicators in each domain used to measure the readiness-adjusted coverage of maternal and newborn care from HHFA data**

| **INIDICATORS PER READINESS** **DOMAIN**  **N=36** | | **Definition** | **HHFA data variable name** |
| --- | --- | --- | --- |
| **Availability of Equipment (12**) | Blood Pressure measuring machine | **“Yes”** for seen available and functional,  “**No”** for (Available but not seen, Not available, Available but not functional, don’t know, missing) | q9320a_18 |
|  | Infant weighing scale | See above | q9320b_21 |
|  | Thermometer | See above | q9320b_29 |
|  | Delivery pack | See above | q9320a_6 |
|  | speculum | See above | q9320b_16 |
|  | Delivery bed | See above | q9320b_2 |
|  | Light source/examination light | See above | q9320b_5 |
|  | Resuscitation table (with heat source) | See above | q9320b_23 |
|  | Newborn bag and mask size 1 | See above | q9325 |
|  | Newborn bag and mask size 0 | See above | q9323 |
|  | incubator | See above | q9320b_24 |
|  | Manual vacuum aspirator | See above | q9320b_14 |
|  | D&C | See above | q9320b_15 |
| **Basic amenities (7)** | Cleaning running water | **“Yes”** for yes, observed,  “**No”** for (yes, reported but not seen, Not available, missing) | q9319_1 |
|  | Hand washing soap | See above | q9319_2 |
|  | Electricity/power source | See above | q12304 |
|  | Emergency transport/Ambulance | “**Yes**” for yes ambulance  “**No**” for other means, No | q2704 |
|  | Functioning toilet | **“Yes”** for yes, observed,  “**No”** for (No, not applicable, missing) | q9335_9 |
|  | computer | “**Yes**” for Yes functional  “**No**” for (yes but not functional, No, missing) | q2501 |
|  | Communication equipment | “**Yes**” for Yes functional  “**No**” for (yes but not functional, No, missing) | q2500 |
| **Staff availability and clinical training (5)** | Staff available 24 hrs. | “**Yes**” for Yes, 24 hours onsite  “**No**” for (Yes, not 24 hours onsite, but not 24 hours on call, No skilled provider available 24 hours) | q9305 |
|  | Guidelines on essential childbirth care | **“Yes”** for yes, observed,  “**No”** for (yes, reported but not seen, Not available, missing) | q9316_1 |
|  | Staff trained in essential child birth care | “**Yes**” for yes “**No**” No | q9318 |
|  | Guidelines on essential newborn care | **Yes”** for yes, observed,  “**No”** for (yes reported but not seen, Not available, missing) | q9802_1 |
|  | Staff trained in -newborn care | “**Yes**” for yes “**No**” (No, there is no policy) | q9803_2 |
| **Tracer drugs and supplies (12)** | Oxytocin | **Yes** for (observed, at least one not expired)  **No** for (Observed available but expired, Not observed reported available, Not observed, not available) | q9333a_10 |
|  | misoprostol | See above | q9333a_9 |
|  | Magnesium sulphate injectable | See above | q9333a_1 |
|  | Calcium gluconate | See above | q12142_4 |
|  | Hydralazine | See above | q12506a_23 |
|  | methyldopa | See above | q12506a_30 |
|  | Ceftriaxone injection | See above | q12501a_10 |
|  | Gentamicin injection | See above | q12501a_18 |
|  | Ampicillin injection | See above | q12501a_5 |
|  | Metronidazole injection | See above | q12501a_21 |
|  | lidocaine | See above | q12143_1 |
|  | Dexamethasone | See above | q9333a_3 |

D&C; Dilation and Curettage

HHFA; Harmonized Health Facility Assessment
