# Supplementary material for: Beyond facility-based births: Is Uganda delivering effective maternal and newborn care? An analysis of the 2022 demographic health survey and 2023 harmonized health facility assessment survey
Source: PLOS Glob Public Health. 2025 Oct 30;5(10):e0004949. doi: 10.1371/journal.pgph.0004949 (PMC12574829; doi:10.1371/journal.pgph.0004949)
Supplement: S3 Table — (DOCX) [file pgph.0004949.s003.docx]

**S3 Table: Summary of DHS data Variables used and their analytical categories**

| **DHS 2022 (Outcome variables)** | | |
| --- | --- | --- |
| **Label** | **UDHS variable name** | **Definition** |
| **Delivered from Health facility**    **Percentage of women who delivered form a health facility in the last years preceding the survey** | b19 ­­_ xx Current age of child in months or months since birth or dead children | b19_01 for **most recent child**  b19_01<=24 **for births within the last 2 years** |
| **1^st^ post-natal check within 48 hrs. for mother** | m63_01 | **Yes**; for minimum-202  **No**; for 203-maximum, missing |
| **Blood pressure was taken during PNC check** | q474a_01 | **Yes**, for yes  **No;** for (No, Don’t know, and missing) |
| **Skin to skin care done** | m77_01 | **Yes**, for yes  **No;** for (No, Don’t know, and missing) |
| **Cord examination** | m78a_01 | **Yes**, for yes  **No;** for (No, Don’t know, and missing) |
| **Counselling on breastfeeding** | m78d_01 | **Yes**, for yes  **No;** for (No, Don’t know, and missing) |
| **Observed breast feeding** | m78e_01 | **Yes**, for yes  **No;** for (No, Don’t know, and missing) |
| **First PNC check within 48hrs for new born** | m75_01 | **Yes**; for minimum-202  **No**; for 203-maximum, missing |
| **Counselling on danger signs of newborn/If baby needs medical attention** | q473c_01 | **Yes**, for yes  **No;** for (No, Don’t know, and missing) |
| **Temperature of newborn measured** | m78b_01 | **Yes**, for yes  **No;** for (No, Don’t know, and missing) |
| **Checked for bleeding** | q474b_01 | **Yes**, for yes  **No;** for (No, Don’t know, and missing) |
|  |  |  |

PNC: Post-natal care

DHS: Demographic Health Survey

UDHS: Uganda Demographic Health Survey

| **UDHS 2022 mothers’ characteristics variable** | | |
| --- | --- | --- |
| **Label** | **UDHS variable name** | **Definition** |
| **Age (in years)** | V012 | <**20**; for (15-190  **20-34**; for (20-24,25-29,30-34)  **35-49**; for (35-39,40-44,45-49) |
| **Marital saltus** | v501 | **Never** **Married**; for (Never in Union)  **Married**; for Married  **Living with partner;** for Living with partner  **Separated;** for widowed, divorced, no longer living together |
| **Education** | V106 | **(kept as recoded in DHS)**  **No education;**  **Primary;**  **Secondary**  **Higher** |
| **Religion** | V130 | **Christian**; Anglican, catholic, Born again, Seventh day Adventist, orthodox  **Muslim**, Muslim  **Others**; for the rest |
| **Residence** | v025 | **(kept as recoded in DHS)**  **Rural**  **Urban** |
| **Household wealth**  **quintile** | v190 | **(kept as recoded in DHS)**  **Lowest Q1,**  **Second Q2,**  **middle Q3,**  **Fourth Q4,**  **Highest Q5** |
| **Type of Health facility** | m15 | See harmonization of facilities (*supplemental table 2)* |
| **Region** | v024 | See harmonization of regions (*supplemental table 2)* |
| **Parity** | v224 | **1 Primipara,**  **1-4 multipara**  **≥5 grand multiparas** |
| **Mode of Childbirth** | m17_1 | **Vaginal delivery**  **Caesarean section** |
| **Sex of the newborn** | b4_01 | **Male**  **Female** |
| **Intention** | V225 | **intended;** Wanted then  **unintended**; wanted later, not at all- |
| **ANC+4** | m14_1 | **0;** No ANC visits  **1-3**  **4+** |
