# Supplementary material for: Beyond facility-based births: Is Uganda delivering effective maternal and newborn care? An analysis of the 2022 demographic health survey and 2023 harmonized health facility assessment survey
Source: PLOS Glob Public Health. 2025 Oct 30;5(10):e0004949. doi: 10.1371/journal.pgph.0004949 (PMC12574829; doi:10.1371/journal.pgph.0004949)
Supplement: Attachment — Submitted filename: Responce to Reviewer comments PLOS_October.docx [file pgph.0004949.s005.docx]

**October 2^nd^, 2025**

Sham Lal

Academic Editor

*PLOS Global Public Health*

**Re : Response Editor and reviewer comments (Manuscript ID : PGPH-D-25-01831)**

Dear Editor,

We sincerely appreciate the opportunity to revise our manuscript titled, *Beyond Facility-Based Births : Is Uganda Delivering Effective Maternal and Newborn Care ? An Analysis of the 2022 Demographic and Health Survey and 2023 Harmonized Health Facility Assessment Survey*, for further consideration for publication in PLOS Global Public Health.

We are grateful for the thoughtful and constructive feedback provided by the editor and reviewers, which has helped to strengthen our work. We have carefully addressed all comments and incorporated the suggested changes into the revised manuscript.

A detailed, point-by-point response outlining the revisions is provided in the pages below.

Thank you for your time and consideration. We hope the revised manuscript meets the journal’s standards and is acceptable for publication

Kind regards,

**Authors**

**Response to Editorial Journal Requirements**

1. Please amend your detailed Financial Disclosure statement. This is published with the article. It must therefore be completed in full sentences and contain the exact wording you wish to be published.

i. State the initials, alongside each funding source, of each author to receive each grant.

ii. State what role the funders took in the study. If the funders had no role in your study, please state: “The funders had no role in study design, data collection and analysis, decision to publish, or preparation of the manuscript.”

**Response 1):** The Financial disclosure statement has now been revised as suggested (page 25, line 589-595) and now reads :

*BT received a PhD scholarship from VLIR-UOS through the MUST-UCOBS project, which also supported his attendance at the DHS course at the Institute of Tropical Medicine (ITM). PMM is supported by a Senior Postdoctoral Fellowship from the Fonds voor Wetenschappelijk Onderzoek (FWO), Belgium (Grant number: 1201925N). The funders had no role in study design, data collection and analysis, decision to publish, or preparation of the manuscript.*

2. Please ensure that your Ethics Statement is available in its entirety at the beginning of your Methods section, under a subheading 'Ethics Statement'**.**

**Response 2):** Ethics statement has been revised and provided in its entirety as follows : (see page 12, lines 328--333)

*This study utilised publicly accessible data and therefore did not require Ethical approval. Authorisation and access to UDHS data were granted by UBOS, while HHFA data access was granted by the Uganda MOH. The UDHS data was accessed from March 27th, 2025, while the HHFA was accessed from May 24th, 2025. The authors were not involved in data collection and did not have access to any identifying information for study participants*

3. Please provide separate figure files in .tif or .eps format.

For more information about figure files please see our guidelines:

<https://journals.plos.org/globalpublichealth/s/figures>

<https://journals.plos.org/globalpublichealth/s/figures#loc-file-requirements>

**Response 3):** Figures have been uploaded as separate files in .tif image format**.**

4. We have noticed that you have uploaded Supporting Information files, but you have not included a list of legends. Please add a full list of legends for your Supporting Information files after the references list.

**Response 4) :** A full list of legends for the supporting information files has been added as suggested **(**page 32, lines 838-845).

5. We note that you have indicated that there are restrictions to data sharing for this study. For studies involving human research participant data or other sensitive data, we encourage authors to share de-identified or anonymized data. However, when data cannot be publicly shared for ethical reasons, we allow authors to make their data sets available upon request. For information on unacceptable data access restrictions, please see <http://journals.plos.org/plosone/s/data-availability#loc-unacceptable-data-access-restrictions>. 
 
 Before we proceed with your manuscript, please address the following prompts:

 a) If there are ethical or legal restrictions on sharing a de-identified data set, please explain them in detail (e.g., data contain potentially identifying or sensitive patient information, data are owned by a third-party organization, etc.) and who has imposed them (e.g., a Research Ethics Committee or Institutional Review Board, etc.). Please also provide contact information for a data access committee, ethics committee, or other institutional body to which data requests may be sent.
 
 b) If there are no restrictions, please upload the minimal anonymized data set necessary to replicate your study findings to a stable, public repository and provide us with the relevant URLs, DOIs, or accession numbers. Please see <http://www.bmj.com/content/340/bmj.c181.long> for guidelines on how to de-identify and prepare clinical data for publication. For a list of recommended repositories, please see <https://journals.plos.org/plosone/s/recommended-repositories>. You also have the option of uploading the data as Supporting Information files, but we would recommend depositing data directly to a data repository if possible.
 
 Please update your Data Availability statement in the submission form accordingly.

**Response 5) :** The data availability statement has been revised and presented below.

*The data used for this study were from the Uganda 2022 Demographic and Health*

*Surveys (DHS) and the 2023 Health Facility Assessment (HHFA). The data were obtained from the Uganda Bureau of Statistics (UBOS) and the Uganda Ministry of*

*Health (MOH). These datasets are not publicly available due to ethical and legal restrictions related to participant confidentiality. However, de-identified data may be made available to interested researchers upon reasonable request from the relevant authorities. Access to the DHS dataset can be requested through the Uganda Bureau of Statistics (UBOS) by contacting* [*info@ubos.org*](mailto:info@ubos.org) *or visiting* [*https://www.ubos.org*](https://www.ubos.org/)*.*

*Access to the HHFA dataset can be requested from the* ***Uganda Ministry of Health (MOH)*** *by contacting* [*ps@health.go.ug*](mailto:ps@health.go.ug)**.**

**Responses to reviewer comments**

1 ) Use of effective coverage cascade terminology and interpretation of findings

1a) In line 135, the order of coverage types provided does not follow the cascade steps, and it's not clear why they would be presented out of order. If the idea is to provide a few exemplars, then I would suggest putting them in order and providing a brief parenthetical definition ; alternatively, replace with a more general description of the cascade from population in need through health service use to health outcomes experienced. More broadly, the introduction should justify the specific focus on input-adjusted and intervention-adjusted coverage in this manuscript and the reason that the calculation of each measure can add value to understanding MNH services in Uganda.

**Response 1a):** Thank you for highlighting the need for clarity in the use and order of effective coverage cascade terminology. We have revised the text to follow the cascade order and have included brief parenthetical definitions to improve readers’ understanding of the concepts (page 5, lines 138-143). We also clarified the rationale for our focus on readiness-adjusted coverage and intervention-adjusted coverage (page 5, lines 143-152).

1b) While it does not become evident until the results section, the measures reported are not strictly within a cascade since intervention coverage is calculated independently of input-adjusted coverage. (Also note that both measures are referred to as effective coverage, while the guidance documents cited suggest using the more specific terminology whenever possible.) The use of intervention coverage on its own without inclusion of readiness should be justified in the methods, with reference to how the use of these two types of measures provides particular insights - why calculate input-adjusted coverage at all if intervention coverage is conceptualized separate from readiness ? Better integration between the two sets of findings would be helpful even if the nested coverage outcome is not calculated - how does knowing which regions have higher readiness but lower intervention coverage, or vice versa, help to target interventions ?

**Response 1b):** Thank you for these comments. We assessed intervention coverage using the target population, without restricting it to women who delivered in "ready facilities." This was because even facilities with poor readiness scores may still be capable of providing certain interventions, and we wanted to capture the coverage of the recommended interventions among the women in need. Our effective coverage measure aimed at assessing not only the percentage of individuals in need of facility-based services who receive them, but also the content(interventions) they received. Our denominator, therefore, included the entire target population, but restricted the interventions to only those who gave birth in health facilities (we considered all non-facility births as not having received any intervention). This approach was proposed and used in other studies <https://doi.org/10.7189/jogh.15.04041>, <https://doi.org/10.1101/2024.12.20.24319361>. Many studies have restricted the linking of DHS data and facility-based data for measuring readiness-adjusted coverage and used only DHS data for intervention coverage <https://doi.org/10.1136/bmjopen-2022-062028>, <https://doi.org/10.1136/bmjopen-2023-077856> . Additionally, many studies focused primarily on a single measure, e.g, readiness-adjusted coverage or intervention coverage. Given that our two data sources were suboptimal for a complete cascade analysis, we set out to use both approaches: determine where the largest gaps are in coverage and propose comprehensive recommendations for both of the gaps, as each has its unique implications.

We agree that this was not a typical cascade. We have attempted to clarify that by showing that the two measures (intervention coverage and readiness-adjusted coverage) were done independently of each other in the methods section (page 10, lines 277-287). We appreciate the need for better integration between the two measures, even though they were not analysed as a strict cascade. We have revised the discussion section, drawing a connection between the two. We highlight how low readiness indicates health system supply gaps (e.g., human resources, drugs, and supplies) while low intervention may indicate gaps in human resource knowledge and skills, poor supervision practices, and overburdened facilities (e.g., work overload). Even without a nested effective coverage, these two measures provide actionable insights and targeted recommendations for regional findings. It provides a dual-lens where both dimensions are put into context (pages 21,22-23, lines 471-480, 519-532).

1c) The difference in readiness between facilities of all types in Kampala vs other regions is quite striking, and surprising given that urban / capital areas often have higher infrastructure and human resources than outlying areas. Is this finding consistent with other studies of the Ugandan health system? Does it have face validity for the author team? Were there indicators that were systematically different in Kampala vs other regions? The findings around deficits in Kampala and the discrepancy between low readiness and higher relative intervention coverage should be interpreted and discussed on together.

**Response 1c):** Thank you for this observation. We agree that while Kampala, being the/capital of Uganda, would often suggest higher readiness, and our study instead found lower readiness.

While it is a commonly held assumption that urban facilities have better readiness than rural facilities, studies from Africa and Asia have shown that this may not always be true (<https://doi.org/10.1186/s12884-023-05643-0> , <https://doi.org/10.1016/j.healthplace.2008.03.004> ) . Several reasons can explain this; one is the fact that these facilities are often characterized by high service volumes as compared to facilities outside Kampala, with only 4.4% of home births in Kampala, as compared to, for example, Karamoja, 19.2% home births. These large volumes of births can overwhelm facilities, leading to stockouts of essential drugs and supplies, the functionality of equipment, referral systems, and human resources. Studies in Uganda have shown that Urban and higher-level facilities are usually more affected by frequent stockouts and lack of basic equipment due to large patient volumes ( <https://doi.org/10.1186/s40545-022-00452-w> , <https://doi.org/10.1186/s40545-022-00412-4> ). There have been recommendations for collaborative action with district authorities within and around the region to equip surrounding facilities and streamline the referral system to address these challenges (<https://doi.org/10.9745/GHSP-D-24-00242>). Additionally, we used indicators for facility-based births that we expect to be available in all facilities offering child birth services, regardless of whether they are lower facilities. We therefore do not include indicators, for example, that are only expected at tertiary facilities that are usually more available in urban settings and private facilities. While urban facilities in Uganda may be more equipped with more advanced equipment like ultrasound scans, Complete Blood Count machines, and theatres, we only assessed indicators that are expected even in rural lower facilities, specifically for essential childbirth care.

We appreciate this suggestion, which has allowed us to strengthen our discussion, and we have incorporated these suggestions into our revised discussion (pages 22- 23, lines 519-532).

We also agree that the relationship between facility readiness and intervention coverage warrants integrated interpretation and discussion. In response, we have revised the discussion section to highlight how regional discrepancies—such as the case of Kampala—reflect a broader pattern observed in previous studies, where facility readiness does not consistently reflect intervention coverage. We have also expanded on potential explanations, including health worker practices and socio-demographic influences, to provide a more nuanced interpretation of the findings (page 21, lines 471-480).

1d) Line 426, the statement ‘The findings also indicate that along the cascade of effective coverage, the largest gap is in the content of care being provided’ is a bit misleading in that 1) the estimates presented are each independent of each other, so it’s not directly a cascade, and 2) the measure used for intervention coverage was more stringent than readiness coverage (all interventions received vs. average readiness score). Presumably if the indicator for facility readiness required all readiness items in place, those scores would drop substantially. The choice to operationalize the measures differently can be justified, but I would keep it in mind - and consider the validity of self-report for intervention indicators - when interpreting the findings.

**Response 1d):** Thank you for pointing this out. This statement has been deleted as it does not reflect our analysis approach. We have also revised our interpretation of findings to correctly reflect our analysis methods (p 20, lines 779-784).

2) Research aim/gap in knowledge: Line 148: The statement on prior research (lack of) regarding MNH effective coverage in Uganda is fairly broad and perhaps not entirely accurate without further qualification. There are certainly closely related studies related to delivery and newborn care (<https://pubmed.ncbi.nlm.nih.gov/34967928/>, <https://pubmed.ncbi.nlm.nih.gov/39151985/>) as well as multiple studies addressing ANC effective coverage, some of which describe this as maternal and newborn care (<https://pubmed.ncbi.nlm.nih.gov/28581379/>, <https://pubmed.ncbi.nlm.nih.gov/30322649/>, <https://pubmed.ncbi.nlm.nih.gov/29632704/>) or that focus on methods (<https://pubmed.ncbi.nlm.nih.gov/29497508/>). Several of these are already cited; I would suggest providing a more specific statement on the gap in the literature - e.g., specific to delivery care, applicable at the national level - so that it is easier to verify this claim.

**Response 2) :** Thank you for this important suggestion. We have revised the statement to specify coverage for facility-based births and also the lack of national-level studies for this area of research (pages 5- 6, lines 157-163).

3 . Methodological clarity: There are a number of gaps and inconsistencies, particularly between the methods section and table headers and footnotes, that make it difficult to tell which procedures were followed and if the findings are accurately presented based on the methods described.

3a) The terminology of parameters within indicators is not standard; the DHS analyses and most quality of care analyses would refer to indicators within domains (e.g., adequate water supply within the infrastructure domain).

**Response 3a):** Thank you for pointing out these inconsistencies. We have revised these terminologies in the main article and supplemental materials**.**

3b). The weighting approach as described in the Methods section does not seem to align with Mallick et al’s weighted average. The Mallick et al approach assigns equal weights by domain regardless of the number of items per domain, in essence, averaging first within domain and then across domains. The approach described here weights each domain by the number of items in it, essentially returning the readiness index to a simple average of all items included. Then in Table 2, two different approaches to defining readiness are described - actually weighting domains evenly (footnote) and using a binary indicator of all readiness items met (column header). Which was actually used (or at least, which was intended to be used)? Please define the actual method used in full in the Methods section, report the corresponding results, and reference back to the selected method only as necessary in the Tables.

**Response 3b):** Thank you for this comment. We indeed notice that the weighting approach we initially described eventually reduces to a simple additive method, making the weighting step redundant. We have revised the methods section to reflect a simple additive method (page 9, lines 259-264). In Table 2, we have revised the sections to reflect the simple additive method. We appreciate your feedback (page 16).

3c) Line 292 - analysis relied on weighted data. More detail is required here: did it also incorporate sampling strata? Was the subpop option used to reflect the fact that the original sample is broader than the population analyzed here? Was weighting used for the HFA as well as DHS data? How were weights utilized in the HFA data, both for regional summaries like Table 2 and for the ecological merging within strata? Typically, weights used for summarizing nationally or regionally are not appropriate for use within strata unless they are appropriately rescaled.

**Response 3c):** We have improved the methods section to clarify that we used the standard DHS survey design and sampling weights in our analysis. We accounted for the multistage stratified sampling design by using cluster identifier (v001) as the primary sampling unit (PSU), the stratification variable (v023), and the DHS-provided weights (v005), which were divided by 1,000,000. We only analyzed the Birth Recode file and restricted the sample to women who had a live birth in the two years preceding the survey and did not use the subpop() option. No survey weights were applied to the HHFA data, either for estimating readiness at the regional level or for linking to the DHS (page 11, lines 309-313).

3d) The methods section does not define the approach for calculating intervention coverage, which it is clear from the results section, was estimated independently from the readiness-adjusted coverage, making it not exactly a nested cascade as was described previously. This may be a justifiable approach, but it requires description and justification in the methods. In general, while it’s helpful to have footnotes on tables for clarity, this information should not add any details that are not reported in the Methods text, but simply express the core idea (e.g., weighted N, or Intervention coverage = crude coverage x self-reported intervention receipt) if needed to orient readers.

**Response 3d):** Thank you for this comment. We have revised the methods section to describe how the intervention coverage was measured (page 10, lines 273-289).

3e) The methods section states that an ecological linkage was conducted based on strata of facilities, but the Results section reports the direct product of regional crude coverage and regional readiness as the input-adjusted coverage figure, without any apparent incorporation of facility type. What happened to the facility strata? Using the figures provided in previous tables, input-adjusted coverage in Kampala would be 40.1% and the Middle East would be 50.2% - not huge differences from the numbers shown, but still different.

**Response 3e):** Thank you for this helpful observation.

We have revised the Methods section to clarify that the ecological linkage was conducted at the regional level only. Our primary objective was to estimate effective coverage at the regional level, in line with ecological linking approaches used in previous studies. Evidence from the literature suggests that regional-level ecological linkage yields comparable estimates to those incorporating facility type or strata (<https://doi.org/10.7189/jogh.08.020803> , <https://pmc.ncbi.nlm.nih.gov/articles/PMC5823029/> ) (page 9, lines 265-269).

**Other**

1) Please update the data availability statement to provide contact information for the request of this information by other research teams. It would also be possible to make fully public the simplified datasets including the readiness indicators and scores and the individuals' facility types.

**Response 1) :** Thank you. The data availability statement has been revised and presented below.

*The data used for this study were from the Uganda 2022 Demographic and Health*

*Surveys (DHS) and the 2023 Health Facility Assessment (HHFA). The data were obtained from the Uganda Bureau of Statistics (UBOS) and the Uganda Ministry of*

*Health (MOH). These datasets are not publicly available due to ethical and legal restrictions related to participant confidentiality. However, de-identified data may be made available to interested researchers upon reasonable request from the relevant authorities. Access to the DHS dataset can be requested through the Uganda Bureau of Statistics (UBOS) by contacting* [*info@ubos.org*](mailto:info@ubos.org) *or visiting* [*https://www.ubos.org*](https://www.ubos.org/)*.*

*Access to the HHFA dataset can be requested from the* ***Uganda Ministry of Health (MOH)*** *by contacting* [*ps@health.go.ug*](mailto:ps@health.go.ug)**.**

2) Line 113 - please provide dates for the MMR estimates

**Response 2):** Thank you for this comment. The dates for the MMR estimates have now been added (page 4, line 114).

3) Line 220: Is there a conceptual or empirical justification for using only most recent of multiples? Would slightly bias effect estimates relative to a target population of births in past 2 years.

**Response 3) :** Thank you for this comment. Our study population was specific to the most recent birth. For multiple births, we restricted the analysis to the last born, as this was the most recent of the births. Additionally, these children share the same birth event and associated maternal responses. Since the mother was the unit of analysis, including all multiples would have introduced duplicate maternal-level data, so we considered the most recent of the multiples**.**

4) Line 297 - I’m not sure that multiplying readiness score by crude coverage needs to be considered a ‘WHO formula’ given that it is a standard component to effective coverage analyses ; if there’s a specific need to reference the WHO formula, the accompanying citations should provide this directly, rather than representing 2 of the many EC analyses that have taken this approach.

**Response 4):** We have now revised this statement to indicate that we adopted an approach used by the cited articles (page 11, line 317).

5) Citation for the delta approach? Please note there is publicly available code for calculating stratified regional effective coverage estimates with variance options that could be applied for this analysis: <https://pmc.ncbi.nlm.nih.gov/articles/PMC7101480/>

**Response 5):** Thank you. This approach has now been cited as suggested (page 11, line 319)**.**

6) Line 319 - 321: 20 - 34 years is a broad swatch of child-bearing women, not sure there’s much value in such a coarse classification unless the goal is to implicitly identify the proportion of women in potentially higher risk adolescent and 35+ age groups, though this could be better achieved by directly stating these %s.

**Response 6) ;** Thank you for this suggestion. We have re-categorised the years and broken down the 20-34 age group. We have additionally revised the age to maternal age at the time of childbirth, which is more appropriate. We had originally reported maternal age at the time of the survey. The new age categories are: ≤19, 20-24, 25-34, and 35-49 (page 13, table 1).

7) Table 1: please include in the table description or footnotes that figures represent weighted data and (presumably) that counts have been rounded to nearest whole number.
   Small query, but I was surprised that 97% of births were not intended - I don’t have the data to check, but the 2016 DHS findings indicated that 59% of births in the past 5 years were wanted at the time of conception. Just curious at this finding and the definition of intended used here to confirm.

**Response 7):** Thank you for these helpful suggestions. We have added footnotes to Table 1 to indicate that the figures represent weighted data and that counts have been rounded to the nearest whole number.

We also appreciate your observation regarding pregnancy intention. Upon review, we identified an error in the categories and variable used to define “intended pregnancy.” This has now been corrected in the revised manuscript and updated accordingly in Table 1, page 13**.**

8) Table 2: To what does the cross-symbol footnote on ‘unweighted percentage availability’ refer? I do not see the symbol in the table or know how to interpret this note.

**Response 8):** Thank you for this observation. This was an error and has now been deleted**.**

9) Line 457 – citation?

**Response 9):** Citations have now been provided (page 21, line 489)**.**

10) Line 422: 86% of women were not lost between crude and intervention - 86% of women did not receive intervention coverage. The drop from 86% crude coverage to 14% intervention coverage is a linear difference of 72% and a relative difference of 84%, though I'm not sure adding additional %s to the interpretation is particularly helpful!

**Response 10) :** Thank you for this observation. There was an error in computing these figures. We also agree that including additional percentage calculations may not necessarily enhance clarity. In response, we have revised the phrase (page 20, lines 447-449)**.**

11) Lines 450 - 466: note there is ongoing discussion of the extent to which government health centers can be reasonably equipped for delivery care and the wisdom of attempting this vs. centralizing delivery care (for instance:[https://pubmed.ncbi.nlm.nih.gov/33055093/](https://pubmed.ncbi.nlm.nih.gov/33055093/), <https://pubmed.ncbi.nlm.nih.gov/33055095/>). This doesn’t have to be a focus of the discussion section, but the findings that such major gaps persist in government health centers does contribute to the broader discussion

**Response 11) :** Thank you for this important suggestion. We agree that this is a critical area of discussion, and we have now addressed it in the revised manuscript discussion section by noting how the persistent gaps we observed in lower-level facilities inform the broader debate around decentralization versus centralization of provision of childbirth care services. We have also cited the recommended references to strengthen this point (page 22, line 498-506)**.**

12) Citation 34 and 44 should be formatted as a journal article, coming through as an internet source

**Response 12) :** These have now been formatted appropriately (see citations 38 and 47)

**Reviewer #2: Overarching comments**

The issues being addressed – effective coverage for maternal newborn care is an extremely important one.
Looking at the title however, and emphasis on ‘facility-based births’, mentioned again in line 152, and ‘childbirth’ and ‘childbirth care’ - mentioned throughout the article, the 10 interventions that were selected (lines 276-280), do not sufficiently cover ‘childbirth care’ especially intrapartum care, a period when mothers and the fetus and newborn are at risk. This has a lot to do with the data sources that were available, used – DHS and HHFA. With that said, the limitation of these data visi a vis the objective, although stated, is not sufficiently acknowledged. In addition, covering other outcomes in particular stillbirths, within limits of data availability and quality, would have added a compelling narrative on quality of care which is the main argument the paper is making, that could have been explored but wasn’t - approximately one half occur during the intrapartum period. Without the emphasis on facility-births alone, the authors could have explored additional indicators such as the coverage targets tracked by Every Woman Every Newborn Everywhere, formerly ENAP/EPMM, such as ANC4+, skilled health personnel at birth, postnatal care, and possibly EmOC from the HHFA.

**Response :** Thank you for this insightful comment. We agree that a more comprehensive measurement of “childbirth care” and “facility-based births” would ideally include additional indicators, especially for intrapartum care. However, our selection of interventions was constrained by our data sources (UDHS 2022 and HHFA), which included limited indicators. We intentionally focused on interventions that occur during the childbirth period, and therefore did not include indicators for effective ANC coverage, such as ANC 4+ attendance or postnatal care coverage, as these fall outside the temporal scope of our study. Nonetheless, we recognize the importance of these services and have recommended that future studies should explore effective coverage for antenatal care and postnatal care as essential components of the continuum of care.

We also agree that including outcomes such as stillbirths would have added value in our effective coverage measurement. However, in line with the effective coverage measurement frameworks we adopted, we did not incorporate outcome-adjusted coverage (<https://doi.org/10.1016/s2214-109x(20)30104-2>). This framework doesn’t recommend the use of outcome-adjusted coverage or linking outcomes to specific interventions or quality domains in maternal and newborn care, due to the multifactorial nature of such maternal and newborn outcomes.

We have now more clearly acknowledged these limitations in the manuscript (page 24, lines 558-562)**.**

Abstract: In the background ‘coverage’ in line 38 should be qualified as the paper is making a point to qualify the different kinds of ‘coverage’. Methods: Lines 53 and 54 (and 60) ‘all 10 recommended interventions’ – I think this has to do less with recommendations, than with data available, and should be qualified as such (related to the overarching comment above). The Conclusion does not sufficiently capture what was studied and is proposed.

**Response :** Thank you. The abstract has been revised appropriately (pages 2-3, lines 37-71)**.**

Introduction : line 105 – should this be ‘newborn’ rather than ‘child’; and qualify SDG target 3.2 as newborn and under-five mortality (line 107). Line 111 – the first month of life is the most vulnerable for ‘newborns’ – did you mean ‘under-fives’? And stillbirths mentioned line 112, (and 117), reinforcing the point make in the overarching comment. Line 113 on maternal mortality – 70 per 100,000 live births being the global target and no country over 140 per 100,000 live births.

**Response :** Thank you for the helpful feedback. The introduction has been revised to incorporate these suggestions. We have clarified the terminology and the SDG targets as recommended (page 4, lines 106-118)**.**

Results and discussion:
Table 1: very struck by the data on ‘pregnancy intention’ ….

**Response :** Thank you for this observation. There was an error in categorising pregnancy intention, and we have corrected these values (page 14, Table 1)**.**

Table 4: in effect the premise of the whole paper, is that having a facility birth (which is what is considered as ‘crude coverage’ means there is a skilled health worker with the resources to carry out the 10 interventions but it is not well explained, and in fact for two of the interventions, the data may also be capturing women who delivered at home and their newborns (post-natal care coverage)

**Response :** Thank you for this important observation. We agree that some of the selected interventions could potentially be received by women who deliver at home. However, for this analysis, and to maintain consistency with our definition of crude coverage (based on facility births), we assumed that only women who delivered in a health facility received the selected interventions. Women who delivered outside a facility were considered not to have received any of the interventions. We have now clarified this assumption in the Methods (page 10, lines 287-289)**.**

Line 387 ‘ten care interventions for childbirth’ – same comment as before

**Response :** We have revised this to indicate that they are selected interventions**.**

Lines 430-436 – in fact the intervention coverage as presented, is also counting ‘contact’ and not ‘content’. For example, several studies have remarked the low quality of counseling that mothers receive – that would have been useful to explore further and/or acknowledge the limitation

**Response :** Thank you for this suggestion. Indeed, there were many interventions for quality of care, such as experience of care (e.g, quality of counselling) that we did not explore in this study. We mainly focused on the provision of care and have added this in our limitations (page 24, lines 558-562)**.**

There are other findings and opportunities that are not sufficiently highlighted/picked up in the discussion such as the encouraging findings for Govt. hospitals, the gaps in readiness for private health facilities – a very significant provider and potential to improve care for millions who are accessing care at high cost at these facilities

**Response:** Thank you for this suggestion. We have incorporated this in our conclusion (page 24, lines 575-580)**.**
